# Adult Combined Heart-Liver Transplantation: The United States Experience

**DOI:** 10.3389/ti.2021.10036

**Published:** 2022-01-04

**Authors:** Sophoclis P. Alexopoulos, W. Kelly Wu, Ioannis A. Ziogas, Lea K. Matsuoka, Muhammad A. Rauf, Manhal Izzy, Roman Perri, Kelly H. Schlendorf, Jonathan N. Menachem, Ashish S. Shah

**Affiliations:** ^1^ Division of Hepatobiliary Surgery and Liver Transplantation, Department of Surgery, Vanderbilt University Medical Center, Nashville, TN, United States; ^2^ Division of Gastroenterology, Hepatology and Nutrition, Department of Medicine, Vanderbilt University Medical Center, Nashville, TN, United States; ^3^ Division of Cardiovascular Medicine, Department of Medicine, Vanderbilt University Medical Center, Nashville, TN, United States; ^4^ Department of Cardiac Surgery, Vanderbilt University Medical Center, Nashville, TN, United States

**Keywords:** liver transplantation, combined heart-liver transplantation, heart transplantation, United Network for Organ Sharing, patient survival

## Abstract

**Background:** We aimed to review the indications and outcomes of adults undergoing combined heart-liver transplantation (CHLT) in the US using national registry data.

**Methods:** Adult (≥18 years) CHLT recipients in the United Network for Organ Sharing database were included (09/1987–09/2020; era 1 = 1989–2000, era 2 = 2001–2010, era 3 = 2011–2020). Survival analysis was conducted by means of Kaplan-Meier method, log-rank test, and Cox regression.

**Results:** We identified 369 adults receiving CHLT between 12/1989–08/2020. The number of adult CHLT recipients (R^2^ = 0.75, *p* < 0.001) and centers performing CHLT (R^2^ = 0.80, *p* < 0.001) have increased over the study period. The most common cardiac diagnosis in the first two eras was restrictive/infiltrative cardiomyopathy, while the most common in era 3 was congenital heart disease (*p* = 0.03). The 1-, 3-, and 5-years patient survival was 86.8, 80.1, and 77.9%, respectively. In multivariable analysis, recipient diabetes [adjusted hazard ratio (aHR) = 2.35, 95% CI: 1.23–4.48], CHLT between 1989-2000 compared with 2011–2020 (aHR = 5.00, 95% CI: 1.13–22.26), and sequential-liver first CHLT compared with sequential-heart first CHLT (aHR = 2.44, 95% CI: 1.15–5.18) were associated with increased risk of mortality. Higher left ventricular ejection fraction was associated with decreased risk of mortality (aHR = 0.96, 95% CI: 0.92–0.99).

**Conclusion:** CHLT is being increasingly performed with evolving indications. Excellent outcomes can be achieved with multidisciplinary patient and donor selection and surgical planning.

## Graphical Abstract

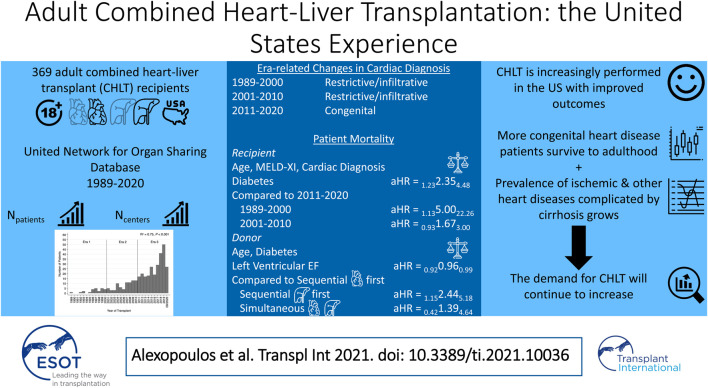

## Introduction

Since the first combined heart-liver transplant (CHLT) in 1984, its indications, patient demographics, and outcomes have evolved significantly [1, 2]. Once a rare and herculean endeavor, CHLT is now being practiced with increasing regularity and improved outcomes [2–7].

The growing practice of CHLT is partially credited to the poor outcomes associated with isolated heart transplantation in the context of concurrent end-stage liver disease. Mortality has been reported to be as high as 50% for patients with known cirrhosis undergoing isolated heart transplantation [8]. In such cases, dual organ transplantation remains the only definitive therapy that can achieve long-term survival. Recent graft survival after CHLT has been found to be similar to that of isolated heart and isolated liver transplantation in carefully selected patients [3].

In the contemporary era, increasing heterogeneity of indications for CHLT as well as variability in listing practices, patient selection, and perioperative management exist. Recipients’ complex pathologies vary broadly—from congenital, ischemic, or infiltrative heart diseases with associated congestive hepatopathy, to liver-based metabolic disorders with systemic complications, to cirrhotic cardiomyopathy [3, 9]. As CHLT becomes an increasingly frequent practice, renewed analyses and review of current practices are necessary to optimize patient selection, perioperative practices, and outcomes. Here, we present a comprehensive retrospective review of adult patients undergoing CHLT in the United States (US) between 1989 and 2020 using national registry data.

## Patients and Methods

### Data Source, Patient Identification, Data Encoding

The United Network for Organ Sharing (UNOS) database administers the Organ Procurement and Transplantation Network under contract with the US Department of Health and Human Services. This database contains data on all transplant candidates listed for solid organ transplantation in the US since October 1987. All data are de-identified, and thus no Institutional Review Board approval was required.

We included all adult (≥18 years) patients undergoing CHLT between September 30th, 1987, and September 4th, 2020, in the US. Patient pre-transplant, transplant, and follow-up data were obtained from the UNOS Standard Transplant Analysis and Research data file (released on September 4th, 2020).

For candidates on dialysis, creatinine was set to 4.0 mg/dl [10]. Estimated Glomerular Filtration Rate (eGFR) was estimated by the 4-variable Modification of Diet in Renal Disease Study equation [eGFR = 175 × (serum creatinine in mg/dL)^1.154^ × (age in years)^−0.203^ × 1.212 (if black) × 0.742 (if female)] [11]. Model for End-stage Liver Disease excluding International Normalized Ratio (MELD-XI) score was calculated as MELD-XI = 11.76*ln(serum creatinine in mg/dL) + 5.112*ln(total bilirubin in mg/dL) + 9.44 [12]. Patients were grouped in the following five cardiac diagnosis subgroups: 1) restrictive/infiltrative cardiomyopathy, 2) ischemic heart disease, 3) congenital heart disease, 4) dilated non-ischemic cardiomyopathy, and 5) other. Transplant sequence was determined by subtracting the cardiac cold ischemia time (CIT) from the liver CIT (<−30 min: sequential-liver first; −30 to 30 min: simultaneous; >30 min: sequential-heart first). Transplant era groups were generated by decade as follows: era 1 = 1989–2000, era 2 = 2001–2010, and era 3 = 2011–2020.

### Statistical Analysis

Categorical variables are described as frequencies and percentages and compared with the chi-square or Fisher’s exact test, while continuous variables are presented as medians [interquartile ranges (IQR)] and compared with the Kruskal-Wallis test. Univariable linear regression was used to assess the number of patients receiving and the number of centers performing CHLT over the study period. Patient survival was the main outcome of interest and was determined as the duration from the date of CHLT until the date of last patient contact or death. Survival analysis was performed using the Kaplan-Meier method, and between-group comparisons were performed with the log-rank test. Cox proportional hazards regression models were fitted to estimate the hazard ratio (HR) and 95% confidence interval (CI). The variables incorporated in the multivariable model were pre-specified to avoid the inferential limitations around selecting covariates for multivariable analysis based on stepwise procedures or univariable comparisons [13]. The multivariable Cox model investigating risk factors of patient mortality in the total cohort included recipient age at transplant, diabetes at listing, MELD-XI score at transplant, cardiac diagnosis, transplant era, donor age, donor diabetes, donor left ventricular ejection fraction, and transplant sequence. The median follow-up time was calculated using the reverse Kaplan-Meier method [14]. To determine the potential impact of annual isolated heart transplant and isolated liver transplant center volume on CHLT outcomes, all centers performing CHLT were classified in tertiles (low, medium, high) based on their isolated heart transplant and isolated liver transplant volume for each given year that each CHLT was performed. All statistical analyses were conducted using Stata IC 16.0 (StataCorp LLC, College Station, TX). All *p*-values were based on two-sided statistical tests, and significance was set at <0.05.

## Results

### Patient Demographics and Clinical Characteristics

**FIGURE 1:**
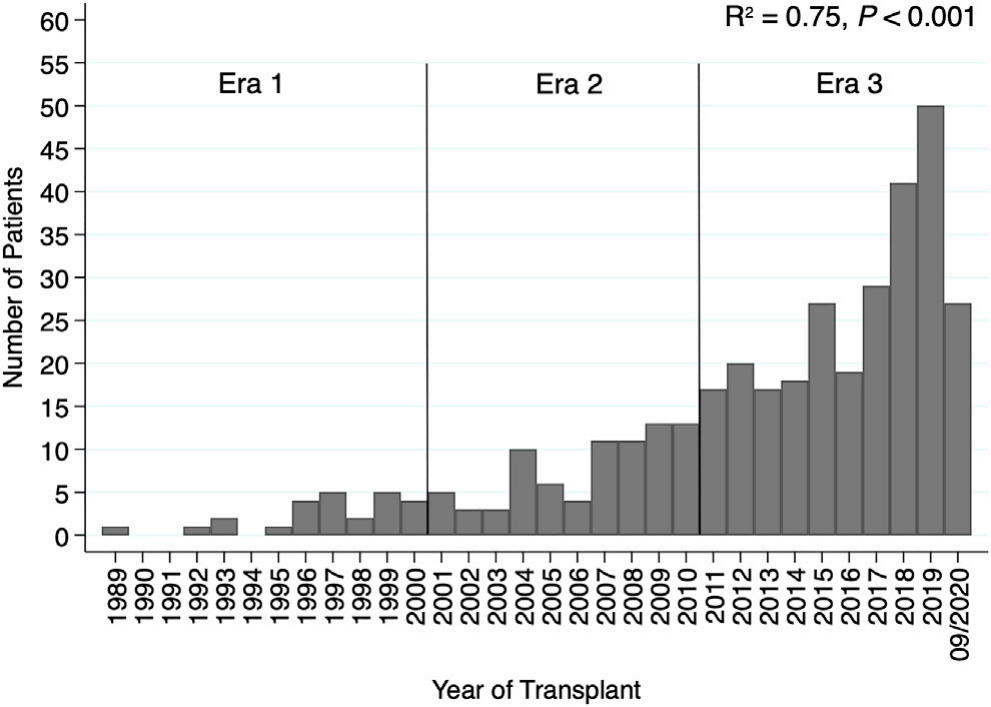
Bar plot demonstrating an increase in the number of patients receiving combined heart-liver transplantation by transplant year.

A total of 369 adult patients who received CHLT between December 1989 and August 2020 were identified. No CHLT recipients were identified between October 1987 and December 1989. The median follow-up time was 49.2 months (95% CI: 42.7–60.9) for the whole cohort. Both the number of adult patients receiving CHLT (R^2^ = 0.75, *p* < 0.001; Figure 1) and the number of centers performing CHLT (R^2^ = 0.80, *p* < 0.001) increased significantly over the study period. The number of centers performing CHLT was 10 between 1989 and 2000, 20 between 2001 and 2010, and 46 between 2011 and 2020.

Several differences in patient characteristics were identified among the three transplant eras. The median MELD score at transplant was higher in patients transplanted between 2011 and 2020 compared with those transplanted between 2001 and 2010 (16.0 vs. 13.5; *p* = 0.007). On the other hand, median MELD-XI was lower in patients transplanted in more recent eras (17.5 vs. 12.4 vs. 11.5; *p* = 0.007). Among the three eras, median waitlist time was significantly shorter in the most recent era (era 1: 86 days vs. era 2: 128 days vs. era 3: 82 days; *p* = 0.045). Cardiac diagnosis was also significantly different among the three eras (*p* = 0.03); the most common cardiac diagnosis in the first two eras was restrictive/infiltrative cardiomyopathy, while the most common cardiac diagnosis in the most recent era was congenital heart disease (CHD). During the first two eras, nearly all CHLTs were sequential-heart first (100 and 97.1%, respectively), while in the most recent era 79.9% were sequential-heart first, 13.8% sequential-liver first, and 6.3% simultaneous (*p* = 0.001). Two donor livers in era 3 underwent machine perfusion. A detailed comparison of patient characteristics among the three transplant eras is depicted in Table 1.

**TABLE 1:** Patient baseline demographic and clinical characteristics by era.

Characteristics	Total (*n* = 369)	1989–2000 (*n* = 25)	2001–2010 (*n* = 79)	2011–2020 (*n* = 265)	*p*-Value
Recipient					
Sex					
Female	113 (30.6%)	8 (32.0%)	19 (25.1%)	86 (32.5%)	0.36
Male	256 (69.4%)	17 (68.0%)	60 (76.0%)	179 (67.6%)	
Age at listing (years)					
Median (IQR)	49.0 (37.0–58.0)	45.0 (27.0–56.0)	48.0 (36.0–56.0)	50.0 (38.0–58.0)	0.30
Age at transplant (years)					
Median (IQR)	49.0 (37.0–58.0)	45.0 (28.0–57.0)	48.0 (36.0–57.0)	50.0 (38.0–58.0)	0.34
Waitlist time (days)					
Median (IQR)	96.0 (36.0–244.0)	86.0 (31.0–350.0)	128.0 (56.0–295.0)	82.0 (35.0–212.0)	0.045
Laboratory MELD score at transplant (*n* = 339)					
Median (IQR)	16.0 (11.0–20.0)	-	13.5 (10.0–19.0)	16.0 (12.0–20.0)	0.007
MELD-XI score at transplant (*n* = 368)					
Median (IQR)	11.9 (8.2–16.3)	17.5 (11.6–22.0)	12.4 (9.0–16.8)	11.5 (7.7–15.5)	0.007
Serum creatinine at transplant (mg/dl) (*n* = 368)					
Median (IQR)	1.2 (0.9–1.6)	1.2 (1.0–2.1)	1.3 (1.0–1.6)	1.2 (0.9–1.6)	0.64
Diabetes at listing (*n* = 355)					
No	295 (83.1%)	17 (89.5%)	65 (87.8%)	213 (81.3%)	0.31
Yes	60 (16.9%)	2 (10.5%)	9 (12.2%)	49 (18.7%)	
Dialysis the week prior to transplant (*n* = 361)					
No	345 (95.6%)	17 (94.4%)	77 (97.5%)	251 (95.1%)	0.62
Yes	16 (4.4%)	1 (5.6%)	2 (2.5%)	13 (4.9%)	
eGFR at transplant (ml/min/1.73 m^2^) (*n* = 368)					
Median (IQR)	61.2 (45.2–81.6)	63.7 (28.7–83.5)	58.9 (45.1–81.9)	62.0 (45.8–81.4)	0.89
CKD stage at transplant (*n* = 368)					
Stage 1	70 (19.0%)	5 (20.8%)	14 (17.7%)	51 (19.3%)	0.01
Stage 2	117 (31.8%)	7 (29.2%)	24 (30.4%)	86 (32.5%)	
Stage 3a	82 (22.3%)	3 (12.5%)	20 (25.3%)	59 (22.3%)	
Stage 3b	62 (16.9%)	2 (8.3%)	11 (13.9%)	49 (18.5%)	
Stage 4	20 (5.4%)	5 (20.8%)	8 (10.1%)	7 (2.6%)	
Stage 5	17 (4.6%)	2 (8.3%)	2 (2.5%)	13 (4.9%)	
BMI at transplant (kg/m^2^) (*n* = 367)					
Median (IQR)	24.5 (21.9–28.3)	23.7 (20.6–27.2)	24.1 (21.1–27.3)	24.9 (21.9–28.6)	0.20
On ventilator at transplant					
No	348 (94.3%)	22 (88.0%)	71 (89.9%)	255 (96.2%)	0.03
Yes	21 (5.7%)	3 (12.0%)	8 (10.1%)	10 (3.8%)	
ICU at transplant (*n* = 365)					
No	201 (55.1%)	15 (60.0%)	53 (67.1%)	133 (51.0%)	0.04
Yes	164 (44.9%)	10 (40.0%)	26 (32.9%)	128 (49.0%)	
Cardiac diagnosis					
Restrictive/infiltrative cardiomyopathy	109 (29.5%)	9 (36.0%)	29 (36.7%)	71 (26.8%)	0.03
Ischemic heart disease	42 (11.4%)	2 (8.0%)	6 (7.6%)	34 (12.8%)	
Congenital heart disease	98 (26.6%)	3 (12.0%)	13 (16.5%)	82 (30.9%)	
Dilated non-ischemic cardiomyopathy	80 (21.7%)	6 (24.0%)	18 (22.8%)	56 (21.1%)	
Other	40 (10.8%)	5 (20.0%)	13 (16.5%)	22 (8.3%)	
Prior cardiac surgery at transplant (*n* = 319)					
No	175 (54.9%)	-	41 (68.3%)	134 (51.7%)	0.02
Yes	144 (45.1%)	-	19 (31.7%)	125 (48.3%)	
VAD at transplant (*n* = 333)					
No	309 (92.8%)	-	67 (93.1%)	242 (92.7%)	0.92
Yes	24 (7.2%)	-	5 (6.9%)	19 (7.3%)	
Cigarette use at listing (*n* = 322)					
No	209 (64.9%)	-	29 (50.0%)	180 (68.2%)	0.009
Yes	113 (35.1%)	-	29 (50.0%)	84 (31.8%)	
Liver diagnosis					
Amyloidosis	74 (20.1%)	7 (28.0%)	19 (24.1%)	48 (18.1%)	<0.001
Cardiac cirrhosis	123 (33.3%)	1 (4.0%)	15 (19.0%)	107 (40.4%)	
NASH	9 (2.4%)	0 (0.0%)	0 (0.0%)	9 (3.4%)	
Alcoholic liver disease	12 (3.3%)	2 (8.0%)	2 (2.5%)	8 (3.0%)	
Other	151 (40.9%)	15 (60.0%)	43 (54.4%)	93 (35.1%)	
Donor					
Age (years)					
Median (IQR)	28.0 (21.0–38.0)	24.0 (17.0–34.0)	30.0 (21.0–43.0)	28.0 (22.0–38.0)	0.04
Donor-to-recipient height ratio (*n* = 366)					
Median (IQR)	1.00 (0.96–1.04)	1.01 (0.97–1.08)	0.99 (0.94–1.03)	1.00 (0.96–1.04)	0.04
Left ventricular ejection fraction (%) (*n* = 343)					
Median (IQR)	62.0 (59.0–65.0)	60.0 (52.5–62.5)	65.0 (56.0–65.0)	61.5 (60.0–65.0)	0.50
Diabetes (*n* = 361)					
No	352 (97.5%)	21 (100.0%)	78 (98.7%)	253 (96.9%)	0.82
Yes	9 (2.5%)	0 (0.0%)	1 (1.3%)	8 (3.1%)	
Liver CIT (hours) (*n* = 352)					
Median (IQR)	7.0 (5.3–8.0)	7.2 (6.7–9.0)	6.7 (6.0–8.0)	7.0 (5.0–8.0)	0.06
Heart CIT (hours) (*n* = 359)					
Median (IQR)	3.0 (2.3–3.8)	2.8 (2.3–3.1)	2.5 (1.9–3.2)	3.1 (2.5–3.9)	<0.001
Transplant sequence (*n* = 344)					
Simultaneous	17 (4.9%)	0 (0.0%)	1 (1.4%)	16 (6.3%)	0.001
Sequential-heart first	291 (84.6%)	20 (100.0%)	68 (97.1%)	203 (79.9%)	
Sequential-liver first	36 (10.5%)	0 (0.0%)	1 (1.4%)	35 (13.8%)	

Abbreviations: CIT, cold ischemia time; CKD, chronic kidney Disease; eGFR, estimated glomerular filtration rate; ICU, intensive care unit; INR, international normalized ratio; IQR, interquartile range; MELD, model for end-stage liver disease; MELD-XI, model for end-stage liver disease excluding INR; NASH, nonalcoholic steatohepatitis; VAD, ventricular-assist device.

Note: Continuous variables are presented as median (interquartile range) and categorical variables as frequencies (%).

Several differences in patient characteristics were identified among the cardiac diagnosis groups. The majority of patients in the restrictive/infiltrative cardiomyopathy, ischemic heart disease, and dilated non-ischemic cardiomyopathy groups were male, while the sex proportions were more equally distributed in the CHD and other groups (*p* < 0.001). The CHD group had lower median age (*p* < 0.001) and MELD-XI at transplant (*p* = 0.01) compared with the other diagnosis groups, and together with the restrictive/infiltrative cardiomyopathy group had longer median waitlist times compared with the other three diagnosis groups (*p* < 0.001). Additionally, as compared with other cardiac diagnosis groups, a higher proportion of the CHD group had undergone prior cardiac surgery at transplant (*p* < 0.001) and had received sequential-liver first and simultaneous CHLT (*p* < 0.001). A detailed comparison of patient characteristics among the five cardiac diagnosis groups is depicted in Supplementary File S1.

### Survival Outcomes

**FIGURE 2:**
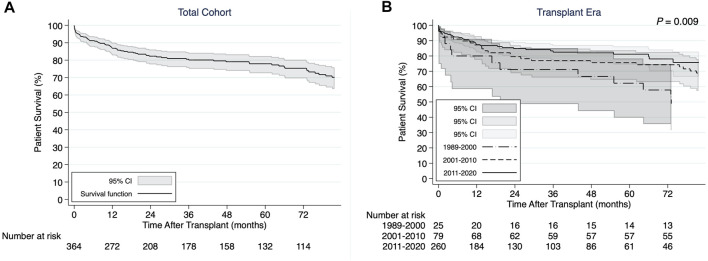
**(A)** Kaplan-Meier patient survival curve for the total cohort of combined heart-liver transplant recipients. **(B)** Kaplan-Meier patient survival curves demonstrating differences by transplant era.

**FIGURE 3:**
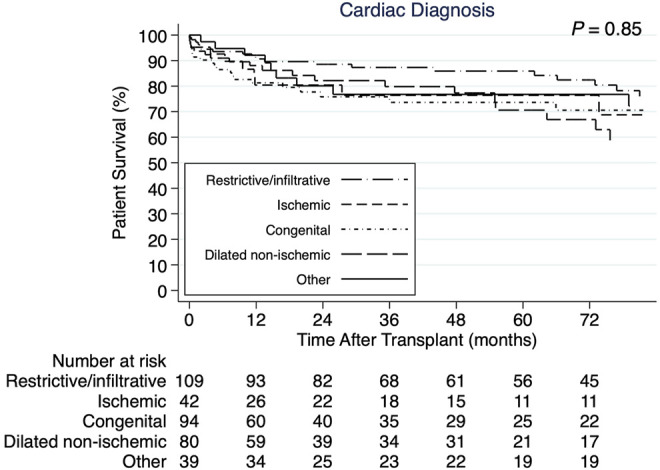
Kaplan-Meier patient survival curves demonstrating no statistically significant difference by cardiac diagnosis.

The 1-, 3-, and 5-years cumulative patient survival point estimates after CHLT for the total cohort were 86.8, 80.1, and 77.9%, respectively (Figure 2A). For those who survived at least 1 year after CHLT (*n* = 286), the 3- and 5-years cumulative patient survival point estimates were 92.6 and 90.3%, respectively. Six patients required liver retransplant over a median post-CHLT period of 19 days (IQR: 13.0–441.0) with indications being hepatic artery thrombosis (*n* = 2), acute rejection (*n* = 1), primary graft failure (*n* = 1), severe preservation injury (*n* = 1), and unknown (*n* = 1). One patient required heart retransplant 12 days post-CHLT due to primary nonfunction. In the total cohort, statistically significant differences in unadjusted patient survival were observed between the three transplant eras (*p* = 0.009; Figure 2B). More specifically, patients undergoing CHLT between 1989 and 2000 demonstrated 2.5 times higher risk of mortality (95% CI: 1.37–4.53; *p* = 0.003) compared with those undergoing CHLT between 2011 and 2020, while no statistically significant differences in survival were observed between those undergoing CHLT between 2001 and 2010 and between 2011 and 2020 (HR = 1.38, 95% CI: 0.85–2.25; *p* = 0.19) (Supplementary File S2). No statistically significant differences were observed in unadjusted patient survival among the cardiac diagnosis groups (*p* = 0.85; Figure 3). In univariable Cox regression analysis (Supplementary File S2), recipient diabetes at listing was associated with an increased risk of patient mortality (HR = 1.72, 95% CI: 1.01–2.94; *p* = 0.047) and higher donor left ventricular ejection fraction with a decreased risk of patient mortality (HR = 0.96, 95% CI: 0.93–0.99; *p* = 0.02). Nevertheless, no statistically significant difference between the groups was determined, when classifying each center performing CHLT as low, medium, or high volume based on either their annual isolated heart transplant (*p* = 0.18) volume or their annual isolated liver transplant volume (*p* = 0.87).

In multivariable Cox regression analysis (Table 2), recipient diabetes at listing (adjusted HR = 2.35, 95% CI: 1.23–4.48; *p* = 0.009), receiving CHLT between 1989 and 2000 compared with 2011–2020 (adjusted HR = 5.00, 95% CI: 1.13–22.26; *p* = 0.03), and receiving sequential-liver first CHLT compared with sequential-heart first CHLT (adjusted HR = 2.44, 95% CI: 1.15–5.18; *p* = 0.02) were associated with an increased risk of patient mortality after CHLT. Higher donor left ventricular ejection fraction was associated with a decreased risk of patient mortality after CHLT (adjusted HR = 0.96, 95% CI: 0.92–0.99; *p* = 0.01).

**TABLE 2:** Multivariable analysis for association among recipient and donor characteristics with patient survival.

Characteristics (n = 312)	Hazard ratio (95% CI)	*p*-value
Recipient		
Age at transplant (years)	0.98 (0.96–1.01)	0.19
Diabetes at listing (ref: no)	2.35 (1.23–4.48)	0.009
MELD-XI score at transplant	1.01 (0.97–1.05)	0.56
Cardiac diagnosis (ref: restrictive/infiltrative cardiomyopathy)		
Ischemic heart disease	0.83 (0.34–1.98)	0.67
Congenital heart disease	0.81 (0.36–1.83)	0.61
Dilated non-ischemic cardiomyopathy	0.88 (0.42–1.84)	0.74
Other	0.57 (0.22–1.49)	0.25
Transplant era (ref: 2011–2020)		
1989–2000	5.00 (1.13–22.26)	0.03
2001–2010	1.67 (0.93–3.00)	0.09
Donor		
Age (years)	1.00 (0.98–1.03)	0.70
Left ventricular ejection fraction (%)	0.96 (0.92–0.99)	0.01
Diabetes (ref: no)	0.97 (0.23–4.13)	0.97
Transplant sequence (ref: Sequential-heart first)		
Simultaneous	1.39 (0.42–4.64)	0.59
Sequential-liver first	2.44 (1.15–5.18)	0.02

Abbreviations: CI, confidence interval; MELD-XI, model for end-stage liver disease excluding INR.

## Discussion

The annual number of adult CHLT in the US has risen sharply, with more CHLT performed during the past 2 years than during either of the previous 2 decades, and a more than four-fold increase over time in the number of centers offering this therapy. While our data demonstrate progressive, era-related improvements in outcomes after CHLT, an appreciation for evolving patient characteristics and indications for CHLT, as well as best practices in surgical techniques, will be critical to ensure appropriate patient selection and favorable outcomes going forward.

Among the most significant changes in CHLT in recent decades has been an evolution in the indications for this procedure. Although restrictive/infiltrative cardiomyopathies secondary to diseases such as amyloidosis and hemochromatosis were the most common indication for CHLT in the early era, CHD is now the most common indication, accounting for nearly one third of CHLT. This trend corresponds to the rising prevalence of liver disease among children with single-ventricle physiology palliated with Fontan. Current life-expectancy post-Fontan exceeds 25 years, by which point many patients develop advanced heart failure and Fontan-associated liver disease manifesting with peri-central and peri-sinusoidal hepatic fibrosis which may progresses to cirrhosis, with increased risk of hepatocellular carcinoma [15, 16]. Even in hemodynamically well-compensated patients, isolated liver transplantation in this population has been ill-advised due to inability to manage elevated right-sided pressures during the anhepatic and reperfusion phases [15]. Despite a progressive era-related increase in use of a sequential-liver first approach for patients with CHD, our data suggest that this approach is associated with worse outcomes.

As would be expected, we identified significant differences in the characteristics of patients undergoing CHLT, based on cardiac diagnosis. Interestingly, however, cardiac diagnosis in and of itself was not associated with differences in post-transplant survival, nor was recipient age, prior tobacco use or MELD-XI score at transplant. Conversely, recipient diabetes, liver-first surgical sequence, and lower donor left ventricular ejection fraction were each independently associated with worse post-CHLT outcomes. These findings underscore the importance of appropriate patient and donor selection for CHLT based on individual patient and donor characteristics, as well as the need for thoughtful pre-operative planning among surgeons from both heart and liver disciplines [12, 17–20]. Despite all of these challenges, the reported survival outcomes of CHLT are similar to those of heart transplant alone [4, 21].

The issue of identification of specific donor factors impacting outcomes persists and may be confounded by changes in donor selection criteria over the years to identify excellent donors, but also a change towards more lenient selection criteria as experience grows. This is supported by the higher donor age, liver and heart CIT, proportion of diabetic donors, as well as by the more optimal donor-recipient height matching and the lower left ventricular ejection fraction in the most recent era. At our center, candidates for CHLT are evaluated jointly by our heart and liver transplant teams and discussed in a multidisciplinary forum that includes transplant cardiologists and hepatologists, adult (and sometimes pediatric) surgeons and, when appropriate, members of the adult CHD team. Upon listing of patients and prior to transplant, surgeons agree on a peri-operative strategy. Team members of both organ programs take part in donor selection. Future research on the optimization of donor selection would enable improved donor-recipient matching. Additionally, the advent and increasing utilization of donor liver machine perfusion may be particularly useful in CHLT as it can mitigate the effects of increased liver graft preservation time while allowing the heart transplant to occur without time pressure constraints [22].

Although the present analysis represents the largest, most comprehensive review of US patients undergoing CHLT during recent decades, certain limitations should be considered when interpreting the results of our study. Due to its retrospective nature, the present study imparts a degree of selection bias regarding patient selection and management. Additionally, there is inconsistency or lack of reporting of parameters that may influence patient survival (i.e., anatomical complexity and number of prior surgeries of the CHD patients, pathologic degree of liver involvement, rationale of performing sequential-liver first CHLT, biliary complications, abortion of liver transplant because of heart transplant induced issues). Lastly, the statistically insignificant results in certain variables may be attributed to lack of power to detect the presence of a potential association.

In conclusion, as more CHD patients survive to adulthood and the prevalence of ischemic and other heart diseases complicated by cirrhosis increases, CHLT will be increasingly necessary to help extend lives. Our data suggest that in the contemporary era, appropriate patient selection for CHLT combined with thoughtful surgical planning and donor selection allow for excellent patient outcomes.

## Data Availability

The data that support the findings of this study are available from the Standard Transplant Analysis and Research file from the United Network for Organ Sharing. Requests to access the datasets should be directed to https://optn.transplant.hrsa.gov/data/request-data/.

## Capsule Sentence Summary

The aim of this paper was to present a comprehensive retrospective review of adult patients undergoing combined heart-liver transplantation (CHLT) in the United States between 1989 and 2020 using national registry data. According to our findings, CHLT is being increasingly performed with evolving indications as more congenital heart disease patients survive to adulthood and the prevalence of ischemic and other heart diseases complicated by cirrhosis increases. Additionally, in the contemporary era, appropriate patient selection for CHLT combined with thoughtful surgical planning and donor selection allow for excellent patient outcomes. Overall, we believe that our work is of increased interest and educational value to the readership of Transplant International and anticipate to decisively influence current perspectives in the field of CHLT.

## Author’s Note

Orally presented at the American Transplant Congress—June 4–9, 2021.

## Abbreviations

CHD, congenital heart disease; CHLT, combined heart-liver transplantation; CI, confidence interval; CIT, cold ischemia time; eGFR, estimated glomerular filtration rate; HR, hazard ratio; QR, interquartile range; MELD-XI, model for end-stage liver disease excluding international normalized ratio; UNOS, United Network for Organ Sharing; US, United States.
