# Supplementary material for: Adult Combined Heart-Liver Transplantation: The United States Experience
Source: Transpl Int. 2022 Jan 4;35:10036. doi: 10.3389/ti.2021.10036 (PMC8842230; doi:10.3389/ti.2021.10036)
Supplement: Supplementary file 1 [file Table1.DOCX]

**Supplemental File 1. Patient Baseline Demographic and Clinical Characteristics by Cardiac Diagnosis**

| **Characteristics** | **Total**  **(n = 369)** | **Restrictive/infiltrative cardiomyopathy**  **(n = 109)** | **Ischemic heart disease**  **(n = 42)** | **Congenital heart disease**  **(n = 98)** | **Dilated non-ischemic cardiomyopathy**  **(n = 80)** | **Other**  **(n = 40)** | ***P*-value** |
| --- | --- | --- | --- | --- | --- | --- | --- |
| **Recipient** |  |  |  |  |  |  |  |
| Sex |  |  |  |  |  |  | < 0.001 |
| Female | 113 (30.6%) | 19 (17.4%) | 7 (16.7%) | 43 (43.9%) | 25 (31.3%) | 19 (47.5%) |  |
| Male | 256 (69.4%) | 90 (82.6%) | 35 (83.3%) | 55 (56.1%) | 55 (68.8%) | 21 (52.5%) |  |
| Age at listing (years) |  |  |  |  |  |  |  |
| Median (IQR) | 49.0 (37.0-58.0) | 58.0 (48.0-62.0) | 56.0 (50.0-60.0) | 32.5 (25.0-41.0) | 50.0 (41.5-57.0) | 45.5 (37.0-53.0) | < 0.001 |
| Age at transplant (years) |  |  |  |  |  |  |  |
| Median (IQR) | 49.0 (37.0-58.0) | 58.0 (49.0-62.0) | 56.0 (50.0-61.0) | 34.0 (26.0-41.0) | 50.5 (42.0-57.0) | 46.0 (38.0-53.0) | < 0.001 |
| Waitlist time (days) |  |  |  |  |  |  |  |
| Median (IQR) | 96.0 (36.0-244.0) | 135.0 (55.0-287.0) | 54.0 (23.0-154.0) | 122.5 (46.0-344.0) | 49.0 (25.5-138.5) | 96.0 (47.5-205.5) | < 0.001 |
| Laboratory MELD score at transplant (n = 339) |  |  |  |  |  |  |  |
| Median (IQR) | 16.0 (11.0-20.0) | 15.0 (11.0-19.0) | 17.0 (9.0-21.5) | 16.0 (11.0-20.0) | 16.0 (10.0-21.0) | 15.0 (10.0-19.0) | 0.71 |
| MELD-XI score at transplant (n = 368) |  |  |  |  |  |  |  |
| Median (IQR) | 11.9 (8.2-16.3) | 12.3 (8.9-16.3) | 12.5 (7.7-16.4) | 10.3 (6.0-14.4) | 12.6 (9.4-19.1) | 12.5 (7.6-16.2) | 0.01 |
| Serum creatinine at transplant (mg/dL) (n = 368) |  |  |  |  |  |  |  |
| Median (IQR) | 1.2 (0.9-1.6) | 1.3 (1.0-1.7) | 1.3 (1.1-1.7) | 1.0 (0.9-1.4) | 1.3 (1.1-1.8) | 1.2 (0.9-1.5) | < 0.001 |
| Diabetes at listing (n = 355) |  |  |  |  |  |  | < 0.001 |
| No | 295 (83.1%) | 88 (85.4%) | 25 (62.5%) | 89 (91.8%) | 58 (75.3%) | 35 (92.1%) |  |
| Yes | 60 (16.9%) | 15 (14.6%) | 15 (37.5%) | 8 (8.3%) | 19 (24.7%) | 3 (7.9%) |  |
| Dialysis the week prior to transplant (n = 361) |  |  |  |  |  |  | 0.02 |
| No | 345 (95.6%) | 106 (99.1%) | 37 (90.2%) | 93 (95.9%) | 71 (91.0%) | 38 (100.0%) |  |
| Yes | 16 (4.4%) | 1 (0.9%) | 4 (9.8%) | 4 (4.1%) | 7 (9.0%) | 0 (0.0%) |  |
| eGFR at transplant (ml/min/1.73 m^2^) (n = 368) |  |  |  |  |  |  |  |
| Median (IQR) | 61.2 (45.2-81.6) | 58.1 (46.6-81.2) | 55.4 (43.5-69.1) | 70.1 (52.6-94.8) | 56.3 (37.6-75.4) | 60.6 (41.7-79.8) | 0.001 |
| CKD stage at transplant (n = 368) |  |  |  |  |  |  | 0.01 |
| Stage 1 | 70 (19.0%) | 17 (15.6%) | 7 (17.1%) | 30 (30.6%) | 9 (11.3%) | 7 (17.5%) |  |
| Stage 2 | 117 (31.8%) | 36 (33.0%) | 9 (22.0%) | 34 (34.7%) | 25 (31.3%) | 13 (32.5%) |  |
| Stage 3a | 82 (22.3%) | 30 (27.5%) | 12 (29.3%) | 17 (17.4%) | 16 (20.0%) | 7 (17.5%) |  |
| Stage 3b | 62 (16.9%) | 18 (16.5%) | 7 (17.1%) | 12 (12.2%) | 15 (18.8%) | 10 (25.0%) |  |
| Stage 4 | 20 (5.4%) | 7 (6.4%) | 2 (4.9%) | 1 (1.0%) | 7 (8.8%) | 3 (7.5%) |  |
| Stage 5 | 17 (4.6%) | 1 (0.9%) | 4 (9.8%) | 4 (4.1%) | 8 (10.0%) | 0 (0.0%) |  |
| BMI at transplant (kg/m^2^) (n = 367) |  |  |  |  |  |  |  |
| Median (IQR) | 24.5 (21.9-28.3) | 25.5 (23.1-28.5) | 26.7 (23.6-30.4) | 22.3 (20.3-26.0) | 24.9 (22.3-30.2) | 23.3 (21.6-27.1) | < 0.001 |
| On ventilator at transplant |  |  |  |  |  |  | 0.75 |
| No | 348 (94.3%) | 104 (95.4%) | 40 (95.2%) | 93 (94.9%) | 75 (93.8%) | 36 (90.0%) |  |
| Yes | 21 (5.7%) | 5 (4.6%) | 2 (4.8%) | 5 (5.1%) | 5 (6.3%) | 4 (10.0%) |  |
| ICU at transplant (n = 365) |  |  |  |  |  |  | 0.16 |
| No | 201 (55.1%) | 62 (56.9%) | 18 (42.9%) | 56 (59.0%) | 39 (48.8%) | 26 (66.7%) |  |
| Yes | 164 (44.9%) | 47 (43.1%) | 24 (57.1%) | 39 (41.1%) | 41 (51.3%) | 13 (33.3%) |  |
| Prior cardiac surgery at transplant (n = 319) |  |  |  |  |  |  | < 0.001 |
| No | 175 (54.9%) | 76 (82.6%) | 23 (60.5%) | 4 (4.3%) | 52 (80.0%) | 20 (64.5%) |  |
| Yes | 144 (45.1%) | 16 (17.4%) | 15 (39.5%) | 89 (95.7%) | 13 (20.0%) | 11 (35.5%) |  |
| VAD at transplant (n = 333) |  |  |  |  |  |  | 0.07 |
| No | 309 (92.8%) | 91 (92.9%) | 37 (92.5%) | 89 (97.8%) | 61 (85.9%) | 31 (93.9%) |  |
| Yes | 24 (7.2%) | 7 (7.1%) | 3 (7.5%) | 2 (2.2%) | 10 (14.1%) | 2 (6.1%) |  |
| Cigarette use at listing (n = 322) |  |  |  |  |  |  | < 0.001 |
| No | 209 (64.9%) | 59 (63.4%) | 16 (41.0%) | 77 (83.7%) | 37 (55.2%) | 20 (64.5%) |  |
| Yes | 113 (35.1%) | 34 (36.6%) | 23 (59.0%) | 15 (16.3%) | 30 (44.8%) | 11 (35.5%) |  |
| Liver diagnosis |  |  |  |  |  |  | < 0.001 |
| Amyloidosis | 74 (20.1%) | 73 (67.0%) | 1 (2.4%) | 0 (0.0%) | 0 (0.0%) | 0 (0.0%) |  |
| Cardiac cirrhosis | 123 (33.3%) | 10 (9.2%) | 9 (21.4%) | 59 (60.2%) | 24 (30.0%) | 21 (52.5%) |  |
| NASH | 9 (2.4%) | 1 (0.9%) | 4 (9.5%) | 0 (0.0%) | 4 (5.0%) | 0 (0.0%) |  |
| Alcoholic liver disease | 12 (3.3%) | 0 (0.0%) | 2 (4.8%) | 0 (0.0%) | 10 (12.5%) | 0 (0.0%) |  |
| Other | 151 (40.9%) | 25 (22.9%) | 26 (61.9%) | 39 (39.8%) | 42 (52.5%) | 19 (47.5%) |  |
| Transplant era |  |  |  |  |  |  | 0.03 |
| 1989-2000 | 25 (6.8%) | 9 (8.3%) | 2 (4.8%) | 3 (3.1%) | 6 (7.5%) | 5 (12.5%) |  |
| 2001-2010 | 79 (21.4%) | 29 (26.6%) | 6 (14.3%) | 13 (13.3%) | 18 (22.5%) | 13 (32.5%) |  |
| 2011-2020 | 265 (71.8%) | 71 (65.1%) | 34 (81.0%) | 82 (83.7%) | 56 (70.0%) | 22 (55.0%) |  |
| **Donor** |  |  |  |  |  |  |  |
| Age (years) |  |  |  |  |  |  |  |
| Median (IQR) | 28.0 (21.0-38.0) | 28.0 (22.0-39.0) | 29.0 (24.0-39.0) | 27.0 (21.0-36.0) | 28.5 (21.5-37.0) | 29.0 (20.0-41.5) | 0.71 |
| Donor-to-recipient height ratio (n = 366) |  |  |  |  |  |  |  |
| Median (IQR) | 1.00 (0.96-1.04) | 0.99 (0.95-1.02) | 1.00 (0.96-1.06) | 1.01 (0.97-1.06) | 1.00 (0.96-1.04) | 1.03 (0.95-1.06) | 0.055 |
| Left ventricular ejection fraction (%) (n = 343) |  |  |  |  |  |  |  |
| Median (IQR) | 62.0 (59.0-65.0) | 60.0 (60.0-65.0) | 64.0 (60.0-65.0) | 62.0 (60.0-68.0) | 60.0 (55.0-65.0) | 65.0 (55.0-67.5) | 0.75 |
| Diabetes (n = 361) |  |  |  |  |  |  | 0.72 |
| No | 352 (97.5%) | 104 (97.2%) | 39 (100.0%) | 95 (97.9%) | 77 (97.5%) | 37 (94.9%) |  |
| Yes | 9 (2.5%) | 3 (2.8%) | 0 (0.0%) | 2 (2.1%) | 2 (2.5%) | 2 (5.1%) |  |
| Liver CIT (hours) (n = 352) |  |  |  |  |  |  |  |
| Median (IQR) | 7.0 (5.3-8.0) | 6.7 (5.1-7.5) | 6.7 (5.6-8.2) | 7.0 (4.4-8.4) | 7.3 (5.6-8.1) | 7.0 (6.0-8.1) | 0.53 |
| Heart CIT (hours) (n = 359) |  |  |  |  |  |  |  |
| Median (IQR) | 3.0 (2.3-3.8) | 2.6 (2.0-3.3) | 3.2 (2.5-3.8) | 3.5 (2.8-4.4) | 2.9 (2.3-3.7) | 2.9 (2.4-3.3) | < 0.001 |
| Transplant sequence (n = 344) |  |  |  |  |  |  | < 0.001 |
| Simultaneous | 17 (4.9%) | 2 (1.9%) | 2 (5.1%) | 9 (9.7%) | 3 (4.1%) | 1 (2.9%) |  |
| Sequential-heart first | 291 (84.6%) | 98 (94.2%) | 34 (87.2%) | 62 (66.7%) | 65 (89.0%) | 32 (91.4%) |  |
| Sequential-liver first | 36 (10.5%) | 4 (3.9%) | 3 (7.7%) | 22 (23.7%) | 5 (6.9%) | 2 (5.7%) |  |

Abbreviations: CIT = cold ischemia time; CKD = Chronic Kidney Disease; eGFR = Estimated Glomerular Filtration Rate; ICU = intensive care unit; INR = international normalized ratio; IQR: interquartile range; MELD = Model for End-stage Liver Disease; MELD-XI = Model for End-stage Liver Disease excluding INR; NASH = nonalcoholic steatohepatitis; VAD = ventricular-assist device.

Note: Continuous variables are presented as median (interquartile range) and categorical variables as frequencies (%).
