# Supplementary material for: Adult Combined Heart-Liver Transplantation: The United States Experience
Source: Transpl Int. 2022 Jan 4;35:10036. doi: 10.3389/ti.2021.10036 (PMC8842230; doi:10.3389/ti.2021.10036)
Supplement: Supplementary file 2 [file Table2.DOCX]

**Supplemental File 2. Univariable Analysis for Association of Recipient and Donor Characteristics with Patient Survival**

| **Characteristics** | **Hazard Ratio (95% CI)** | ***P*-value** |
| --- | --- | --- |
| **Recipient** |  |  |
| Male sex (ref: female) | 0.99 (0.64-1.54) | 0.98 |
| Age at listing (years) | 1.00 (0.98-1.01) | 0.69 |
| Age at transplant (years) | 1.00 (0.98-1.01) | 0.78 |
| Waitlist time (days) | 1.00 (0.99-1.00) | 0.09 |
| Laboratory MELD score at transplant | 1.00 (0.96-1.03) | 0.81 |
| MELD-XI score at transplant | 1.01 (0.98-1.04) | 0.59 |
| Serum creatinine at transplant (mg/dL) | 1.10 (0.86-1.41) | 0.44 |
| Diabetes at listing (ref: no) | 1.72 (1.01-2.94) | 0.047 |
| Dialysis the week prior to transplant (ref: no) | 2.09 (0.91-4.81) | 0.08 |
| eGFR at transplant (ml/min/1.73 m^2^) | 1.00 (0.99-1.01) | 0.28 |
| CKD stage at transplant (ref: Stage 1) | - | - |
| Stage 2 | 0.86 (0.47-1.58) | 0.63 |
| Stage 3 | 0.97 (0.55-1.71) | 0.92 |
| Stage 4-5 | 1.03 (0.50-2.12) | 0.93 |
| BMI at transplant (kg/m^2^) | 1.02 (0.97-1.06) | 0.51 |
| On ventilator at transplant (ref: no) | 1.02 (0.47-2.23) | 0.95 |
| ICU at transplant (ref: no) | 0.90 (0.60-1.36) | 0.63 |
| Cardiac diagnosis (ref: restrictive/infiltrative cardiomyopathy) | - | - |
| Ischemic heart disease | 1.44 (0.72-2.89) | 0.31 |
| Congenital heart disease | 1.23 (0.71-2.14) | 0.47 |
| Dilated non-ischemic cardiomyopathy | 1.26 (0.72-2.20) | 0.42 |
| Other | 1.13 (0.56-2.27) | 0.73 |
| Prior cardiac surgery at transplant (ref: no) | 1.42 (0.86-2.33) | 0.17 |
| VAD at transplant (ref: no) | 1.07 (0.43-2.67) | 0.88 |
| Cigarette use at listing (ref: no) | 1.30 (0.79-2.14) | 0.30 |
| Liver diagnosis (ref: Amyloidosis) | - | - |
| Cardiac cirrhosis | 1.04 (0.60-1.81) | 0.89 |
| NASH | 2.14 (0.64-7.15) | 0.22 |
| Alcoholic liver disease | 1.45 (0.50-4.20) | 0.49 |
| Other | 0.82 (0.49-1.38) | 0.46 |
| Transplant era (ref: 2011-2020) | - | - |
| 1989-2000 | 2.50 (1.37-4.53) | 0.003 |
| 2001-2010 | 1.38 (0.85-2.25) | 0.19 |
| **Donor** |  |  |
| Age (years) | 1.01 (0.99-1.02) | 0.47 |
| Donor-to-recipient height ratio | 2.00 (0.08-48.51) | 0.67 |
| Left ventricular ejection fraction (%) | 0.96 (0.93-0.99) | 0.02 |
| Diabetes (ref: no) | 0.91 (0.22-3.70) | 0.89 |
| Liver CIT (hours) | 1.01 (0.93-1.10) | 0.78 |
| Heart CIT (hours) | 1.09 (0.90-1.33) | 0.36 |
| Transplant sequence (ref: Sequential-heart first) | - | - |
| Simultaneous | 1.22 (0.38-3.91) | 0.73 |
| Sequential-liver first | 1.63 (0.84-3.19) | 0.15 |
| Liver-heart CIT difference (hours) | 0.98 (0.90-1.07) | 0.64 |

Abbreviations: CI = confidence interval; CIT = cold ischemia time; CKD = Chronic Kidney Disease; eGFR = Estimated Glomerular Filtration Rate; ICU = intensive care unit; INR = international normalized ratio; MELD = Model for End-stage Liver Disease; MELD-XI = Model for End-stage Liver Disease excluding INR.
